# Abnormal body composition, cardiovascular endurance, and muscle strength in pediatric SLE

**DOI:** 10.1186/s12969-016-0110-8

**Published:** 2016-09-05

**Authors:** Sangeeta Sule, Kevin Fontaine

**Affiliations:** 1 Department of Pediatrics, Johns Hopkins University, 200 N. Wolfe Street, Suite 2126, Baltimore, MD 21287 USA; 2 University of Alabama at Birmingham School of Public Health, 1720 2nd Avenue South, RPHB 214C, Birmingham, AL 35294 USA

## Abstract

**Background:**

Children with SLE are known to have higher fat mass compared to their peers but there are no published data regarding exercise capacity as measured by cardiovascular endurance and muscle strength. In our pilot study of ten children with SLE, we sought to examine body composition, cardiovascular endurance, and isometric muscle strength.

**Findings:**

Ten pediatric SLE patients were studied with a mean age of 15.5 years and 90 % female. Percent body fat above 30 %, the recommended normal level in adolescent females, were found in 89 % of female subjects and 40 % of all participants had lower than the recommended norms of muscle mass for their age/gender. Subjects with renal disease were more likely to have low muscle mass compared to those without renal disease (*p* = 0.03). Cardiovascular endurance was reported as estimated maximal oxygen consumption (VO_2_max) during cycle ergometry. All participants scored in less than fifth percentile for VO_2_max measurements compared to data from age and gender matched published norms. Isokinetic muscle strength testing was performed on upper and lower extremities. Only one participant (male subject) reached goal percentiles for age and gender.

**Conclusions:**

We found significant deficit in body composition, muscle strength and cardiovascular endurance in the pediatric SLE population compared to reported published norms. Clinicians should consider these data and focus on exercise programs that can increase aerobic capacity and muscle strength in this high-risk population.

## Background

Patients with systemic lupus erythematosus (SLE) are considered to be at higher risk for early atherosclerosis and coronary artery disease (CAD), independent of traditional risk factor such as hypertension and smoking [1–6]. In order to lessen this risk, we encourage our patients to engage in exercise and physical activity. This increased activity may also improve overall quality of life, fatigue, pain, and emotional well-being [7].

SLE patients may experience difficulties when trying to become more active; for example, adults with SLE are known to have decreased cardiovascular endurance, lower muscle strength, and lower lean muscle mass compared to age-matched controls [8, 9]. Children with SLE are known to have higher fat mass compared to their peers; lower lean muscle mass in these children has been associated with complications such as vertebral fractures [10–14]. In our pilot study, we sought to examine body composition, cardiovascular (CV) endurance, and isometric muscle strength in ten pediatric patients with SLE.

## Findings

### Subjects

Institutional review board (IRB) approval was obtained. Guardians were consented and youth were assented for participation in the study. The participants were recruited from consecutive pediatric SLE patients, defined as age ≤18 years at SLE onset, seen at the Johns Hopkins Children’s Center. We excluded patients with active arthritis or active myositis because the participants needed to be able to pedal without restrictions on the cycle ergometry for assessment of CV endurance. Participants with a past history of either arthritis or myositis were permitted. Of the 15 consecutive pediatric SLE patients, four had active arthritis and one had active myositis, leaving ten pediatric SLE patients in this pilot study. All patients met the American College of Rheumatology (ACR) criteria for SLE [15, 16].

### Measures

At study entry, demographic data including age at enrollment, age at lupus diagnosis, gender, and race, were collected. Clinical and laboratory data were collected at the participant’s outpatient rheumatology clinic appointment within 4 weeks of the study visit and were reported as present if seen at that visit. Systemic lupus erythematosus disease activity index scores (SLEDAI) scores were calculated based on these data [17].

We assessed perception of physical fitness by asking participants about their daily physical activity and whether they would consider themselves physically active over the past year. We assessed the duration in minutes for each physical activity per week, not including required school gym participation.

Total body lean and fat mass were assessed by assessed by dual energy X-ray absorptiometry (DEXA) (GE Lunar Prodigy, Software V. 13). Body weight and height were obtained by measurement at the time of the study visit. Patients were divided into age-adjusted categories for overweight and obese. Definitions for normal ranges of BMI were per the Centers for Disease Control and Prevention (CDC) guidelines: normal; <85^th^ percentile; overweight, 85–95^th^ percentile; obese, >95^th^ percentile. The CDC recommended levels for adolescents are <30 % of total body composition body fat and >60 % lean muscle mass.

CV endurance capacity was assessed using peak oxygen uptake (VO_2_ peak) during cycle ergometry. A Cardinal Health Vmax metabolic system was used to assess oxygen uptake while the participants pedaled on the stationary bicycle. The initial workload was set at 0 W and was increased by 20 W every 3 min until participants noted fatigue. Normal values were obtained from published reference data in adolescents and varied based on age and gender of the comparative SLE participant [18–21].

A Biodex unit was used to measure isometric muscle strength using peak isokinetic torques of elbow extension and flexion at 60 and 120 degrees/second, adjusted for body weight. Normative elbow values were obtained from reference data published by Biodex and ranged from 23 to 30 % peak torque/body weight for 60 degrees/second and 13–24 % peak torque/body weight for 120 degrees/second. Knee peak isokinetic torques were obtained at 60 and 180 degrees/second. Normal knee ranges were 80–95 % peak torque/body weight for 60 degrees/second movement and 50–65 % peak torque/body weight for 180 degrees/second movement.

### Statistical analyses

We performed two-sided, unpaired t-tests for comparison of means and Fisher’s exact tests for comparison of groups between our cohort and published reference data for categorical body composition data, continuous CV endurance data, and continuous muscle strength data [18, 20, 22]. Simple linear regression analyses were used to determine predictors of body composition and CV endurance via VO2 peak during cycle ergometry. We did not attempt multiple regression for these analyses as there were too few variables/subjects. Significance levels were set at a *p*-value <0.05.

### Results

Demographic characteristics are shown in Table 1. Self-reported physical activity was high in our cohort. Eighty percent of patients reported that they would consider themselves physically active over the past year. Sixty percent noted that they participated in physical activity 1–3 days per week for 30–60 min, 20 % participated 4–6 days/week for 0–30 min, and 20 % no exercise.

**Table 1:** Patient characteristics at study entry

Characteristic	Value
Age, mean (SD)	15.5 (3.1)
Female gender, *n* (%)	9 (90 %)
African American, *n* (%)	8 (80 %)
Mean years with SLE, (SD)	4.2 (2.3)
Malar rash, *n* (%)	6 (60 %)
Kidney disease, *n* (%)	5 (50 %)
Focal proliferative, *n* (%)	1 (10 %)
Membranous, *n* (%)	1 (10 %)
Diffuse proliferative, *n* (%)	3 (30 %)
ANA positive, *n* (%)	10 (100 %)
dsDNA positive, *n* (%)	9 (90 %)
Ro/La positive, *n* (%)	2 (20 %)
Smith positive, *n* (%)	3 (30 %)
Low C3/C4, *n* (%)	2 (20 %)
Elevated ESR/CRP, *n* (%)	0 (0 %)
Anemia, *n* (%)	0 (0 %)
Prednisone	4 (40 %)
Dose	0.1–0.5 mg/kg/day
Duration, mean months, (SD)	2 (3.1)
Hydroxychloroquine, *n* (%)	10 (100 %)
Methotrexate, *n* (%)	2 (20 %)
Mycophenolate mofetil, *n* (%)	4 (40 %)

Fifty percent of participants were above recommended BMI guidelines. Mean BMI was 23.7 kg/m2 (range 17.1–31.3 kg/m2). Five subjects had BMI within the normal range, three were classified as overweight, and two were obese.

Eighty nine percent of female subjects had a body percent fat above 30 %. Mean body fat percent was elevated in the entire cohort with a mean of 36.7 % (range in all 9–50.7 %), and mean of 39.7 % in the nine females (range 25.7–50.7 %). Lean muscle mass ranged from 49.2 to 91 % with a mean of 63.3 %. Forty percent of participants had lower than 60 % muscle mass for their age/gender. These same four subjects were also noted to have elevated body fat (>=30 %). We performed exploratory analyses to identify factors associated with abnormal body composition (Table 2). Participants with renal disease were more likely to have low muscle mass compared to those without renal disease (*p* = 0.04). The presence of Smith antibody was associated with higher muscle mass (*p* = 0.03).

**Table 2:** Body composition by dual energy X-ray absorptiometry (DEXA) scan

Variable	≥30 % Body Fat	<30 % Body Fat	*P*-Value	≤60 % Lean Muscle Mass	>60 % Lean Muscle Mass	*P*-value
	*N* = 8	*N* = 2		*N* = 4	*N* = 6	
Mean Age, years ± Standard deviation (SD)	16.3 ± 2.7	12.5 ± 3.5	0.4	15.7 ± 4	15.3 ± 2.7	0.8
African American, n	6	2	0.4	4	4	0.2
Years of SLE ± SD	4.1 ± 2.5	4.5 ± 0.7	0.7	3.3 ± 1.6	5.5 ± 2.6	0.2
Physically Active in the past year						
Yes	6	2	0.9	2	6	0.1
No	2	0		2	0	
Malar rash						
Present	6	0	0.1	4	2	0.07
Absent	2	2		0	4	
Kidney Disease						
Present	4	1	0.9	4	1	0.04
Absent	4	1		0	5	
dsDNA Antibody						
Present	8	1	0.2	4	5	0.9
Absent	0	1		0	1	
Ro/La Antibodies						
Present	2	0	0.9	1	1	0.9
Absent	6	2		3	5	
Smith Antibody						
Present	3	0	0.9	3	0	0.03
Absent	5	2		1	6	
Low C3/C4						
Present	2	0	0.9	2	0	0.1
Absent	6	2		2	6	
Mean SLEDAI, ± SD	1.3 ± 1.1	0	0.06	2 ± 1.4	0.4 ± 0.2	0.07
Prednisone						
Taking	4	0	0.4	3	1	0.1
Not taking	4	2		1	5	
Cumulative prednisone dose, mg (range)	900 (0–3600)	0	0.1	1725 (0–3600)	50 (0–300)	0.1
Hydroxychloroquine						
Taking	8	2	--	4	6	--
Not taking						
Methotrexate						
Taking	2	0	0.9	1	1	0.9
Not taking	6	2		3	5	
Mycophenolate mofetil						
Taking	3	1	0.9	2	2	0.9
Not taking	5	1		2	4	

CV endurance was reported as estimated maximal oxygen consumption (VO_2_max). All participants had low VO_2_max measurements compared to data from age and gender matched normal controls. Eighty percent of subjects scored at <2^nd^ percentile compared to healthy controls while the remaining 20 % of SLE subjects scored between the 2^nd^ and 5^th^ percentile. In regression analysis, the presence of kidney disease was associated with worse VO_2_max measurements (Coef: −10, *p* = 0.007) (Table 3).

**Table 3:** Regression analysis of cardiovascular endurance

Variable	Coefficient	*P*-value
Age, years	0.5	0.5
African American Race	−9.7	0.06
Years of SLE	0.2	0.06
Physically Active	7.7	0.2
Malar rash	−5.6	0.2
Kidney Disease	−10	**0.007**
dsDNA positive,	−11.6	0.1
Ro/La positive	−1.5	0.8
Smith positive	−9.9	**0.02**
Low C3/C4	−10.4	**0.04**
Prednisone	−9.1	**0.02**
Methotrexate	−1.5	0.8
Mycophenolate mofetil	−6.5	0.1
Body Fat >30 %	−5.4	0.3
Muscle mass >60 %	9.7	**0.01**

Isometric muscle strength testing was performed on dominant elbow and knee. In elbow testing, at 60 degrees/second, only one participant reached goal percentiles (male subject). All other participants were significantly weaker than normal controls, ranging from 10 to 20 % below goal. At 120 degrees/second, none of the participants reached published goals; however, the difference between actual to goal results was much closer (1–10 %). With knee strength testing, no SLE participant reached normal goal values (deficit 2–58 % at 60 degrees/second and deficit 10–30 % at 180 degrees/second).

## Conclusions

In this study, we found significant deficits in muscle strength and CV endurance. These data are in line with previous studies that have found that adults with SLE have diminished muscle and aerobic capacity compared to age-matched controls [8, 9].

Body composition is known to negatively affect quality of life in SLE. In a study of 202 pediatric SLE patients, investigators compared health related quality of life between obese, defined as BMI ≥95^th^ percentile for age and gender, and non-obese patients [14]. Obese pediatric SLE patients had significantly worse school function, impaired physical function, and significantly more pain/hurt compared to non-obese pediatric SLE patients and historical normal controls [14]. In a study of 138 adult women with SLE, obesity as defined by DEXA scan was associated with significant cognitive impairment and decreased executive function, although the mechanism for this relationship is not completely clear [23].

Cardiovascular endurance has been measured in adults with SLE. In 34 women with SLE, lower aerobic capacity was found in patients compared to controls [9]. There was no correlation between CV endurance and disease activity or medications. In our cohort, CV endurance was significantly decreased, with all of our subjects testing below the 5^th^ percentile of normal children. This low CV endurance could put children with SLE at increased risk of cardiovascular disease.

Some participants in our study had normal muscle mass, but muscle strength was decreased in the majority. In adults with SLE, both isometric and dynamic muscle strength has been shown to be decreased compared to healthy adults. In a study of 146 women with SLE, muscle mass did not correlate with muscle strength measurements, and reduced lower extremity muscle strength was associated with worse self-reported physical disability [24]. In a longitudinal study of these same women, muscle measurements were repeated approximately 2 years apart [25]. Reduced lower extremity muscle strength predicted clinically significant decreases in physical function, particular among the weakest subjects. Our findings are particularly worrisome for the pediatric SLE population as significant deficits in muscle strength could lead to reduced physical function and activity.

There are some limitations to our study including small sample size, cross-sectional analysis, and exclusion of SLE subjects with active arthritis or myositis. The subjects in our cohort had relatively low disease activity and this may bias our findings, particularly in body composition associations. We used published normative values for control data and this lack of a local comparative group may be a weakness. This is a small sample size and the regression analyses should be interpreted with caution and used as hypothesis-generating for future studies. The strengths of our study include complete body composition, exercise capacity, and muscle strength testing data obtained during the same visit.

Our study reinforced published data of increased body fat and low muscle mass in pediatric patients with SLE. Despite low SLE disease activity, the subjects in our cohort had significant deficits in CV endurance and muscle strength. Clinicians should consider focusing on exercise programs that can increase aerobic capacity in this high-risk population.

## Abbreviations

ACR: American College of Rheumatology
BMI: Body mass index
CAD: Coronary artery disease
CDC: Centers for disease control and prevention
CV: Cardiovascular
DEXA: Dual energy x-ray absorptiometry
dsDNA: Double-stranded deoxyribonucleic acid
SD: Standard deviation
SLE: Systemic lupus erythematosus
SLEDAI: Systemic lupus erythematosus disease activity index scores
VO_2_ max: Estimated maximal oxygen consumption
